# Performance and utility of more highly sensitive malaria rapid diagnostic tests

**DOI:** 10.1186/s12879-021-07023-5

**Published:** 2022-02-04

**Authors:** Hannah C. Slater, Xavier C. Ding, Sophia Knudson, Daniel J. Bridges, Hawela Moonga, Neil J. Saad, Martin De Smet, Adam Bennett, Sabine Dittrich, Laurence Slutsker, Gonzalo J. Domingo

**Affiliations:** 1 Diagnostics Program, PATH, Seattle, WA USA; 2 Malaria and Neglected Tropical Diseases, PATH, Seattle, WA USA; 3 Foundation for Innovative New Diagnostics, Geneva, Switzerland; 4 PATH Malaria Control and Elimination Partnership in Africa (MACEPA), Lusaka, Zambia; 5 National Malaria Elimination Centre, Zambia Ministry of Health, Lusaka, Zambia; 6 Médecins Sans Frontières, Phnom Penh, Preah Vihear Cambodia; 7 Médecins Sans Frontières, Brussels, Belgium; 8 Malaria Elimination Initiative, Global Health Group, University of California San Francisco, San Francisco, CA USA

**Keywords:** HS-RDT, Rapid diagnostic test, Malaria diagnosis, Cross-sectional surveys

## Abstract

**Background:**

A new more highly sensitive rapid diagnostic test (HS-RDT) for *Plasmodium falciparum* malaria (Alere™/Abbott Malaria Ag P.f RDT [05FK140], now called *NxTek*™* Eliminate Malaria Ag Pf*) was launched in 2017. The test has already been used in many research studies in a wide range of geographies and use cases.

**Methods:**

In this study, we collate all published and available unpublished studies that use the HS-RDT and assess its performance in (i) prevalence surveys, (ii) clinical diagnosis, (iii) screening pregnant women, and (iv) active case detection. Two individual-level data sets from asymptomatic populations are used to fit logistic regression models to estimate the probability of HS-RDT positivity based on histidine-rich protein 2 (HRP2) concentration and parasite density. The performance of the HS-RDT in prevalence surveys is estimated by calculating the sensitivity and positive proportion in comparison to polymerase chain reaction (PCR) and conventional malaria RDTs.

**Results:**

We find that across 18 studies, in prevalence surveys, the mean sensitivity of the HS-RDT is estimated to be 56.1% (95% confidence interval [CI] 46.9–65.4%) compared to 44.3% (95% CI 32.6–56.0%) for a conventional RDT (co-RDT) when using nucleic acid amplification techniques as the reference standard. In studies where prevalence was estimated using both the HS-RDT and a co-RDT, we found that prevalence was on average 46% higher using a HS-RDT compared to a co-RDT. For use in clinical diagnosis and screening pregnant women, the HS-RDT was not significantly more sensitive than a co-RDT.

**Conclusions:**

Overall, the evidence presented here suggests that the HS-RDT is more sensitive in asymptomatic populations and could provide a marginal improvement in clinical diagnosis and screening pregnant women. Although the HS-RDT has limited temperature stability and shelf-life claims compared to co-RDTs, there is no evidence to suggest, given this test has the same cost as current RDTs, it would have any negative impacts in terms of malaria misdiagnosis if it were widely used in all four population groups explored here.

**Supplementary Information:**

The online version contains supplementary material available at 10.1186/s12879-021-07023-5.

## Background

Rapid diagnostic tests (RDTs) and microscopy are the cornerstone of confirmation of clinical malaria diagnosis in most endemic countries and are also widely used for prevalence surveys. They also have more limited use in other scenarios, including active and reactive case detection and screening pregnant women. RDTs have high sensitivity against clinical infections for *Plasmodium falciparum* [1, 2] as these are typically associated with higher parasite densities and thus higher levels of antigenemia. However, sensitivity when used for detecting asymptomatic infections is considerably lower.

Recently, a *Plasmodium falciparum* histidine-rich protein 2 (HRP2)–based RDT (Alere™/Abbott Malaria Ag P.f RDT [05FK140], now called *NxTek™ Eliminate Malaria Ag Pf*) with a tenfold improved analytical sensitivity as compared to average conventional RDTs (co-RDTs) was prequalified by the World Health Organization (WHO). From here on, we refer to this test as the highly sensitive RDT (HS-RDT).

An unprecedented wide range of studies has been conducted using the HS-RDT in a variety of transmission settings and use cases to investigate the practical benefits of its lower limit of detection (LOD). First and foremost, this test was intended to identify asymptomatic infections in mass screening and active case detection interventions, particularly in low-transmission settings. The diagnostic sensitivity of an RDT is driven primarily by two factors: (i) LOD of the test (i.e., its analytical sensitivity) and (ii) the malaria antigen distribution in the sampled infected population. It has been shown that conventional RDTs are more sensitive in high-transmission settings [3], which is most likely because, on average, individuals have higher parasite densities and thus higher antigenemia [4]. To assess the utility of the HS-RDT, we need to better understand the how it performs in comparison to co-RDTs or more sensitive nucleic acid amplification–based tests (NAATs) (e.g., polymerase chain reaction [PCR]) and in different transmission settings.

Questions remain around the utility of more highly sensitive RDTs for other use cases: for example, given that current RDTs perform well in clinical settings, will a more sensitive test increase the number of people with symptomatic malaria being correctly diagnosed? There may be individuals that test positive due to having chronic asymptomatic infections, but malaria is not the primary cause of their fever. Furthermore, given that HRP2 decays relatively slowly after parasite clearance, there is a risk that HS-RDTs will increase the numbers of false positives in individuals with recently cleared infections. Twenty-five percent of co-RDTs are estimated to remain positive for at least 20 days after the clearance of parasites, and this is expected to be greater for a more sensitive RDT [5].

There has been interest in testing pregnant women for malaria during antenatal care (ANC) visits, where drugs more effective than those used in intermittent preventative therapy during pregnancy (IPTp) could be given upon testing positive [6]. *P. falciparum* infections are typically harder to detect in pregnant women as the parasites commonly sequester in the placenta [7] and treating asymptomatic infections has been shown to have positive impacts on both the mother and the infant [8]. Therefore, more sensitive diagnostics to identify pregnant women to be treated and cleared of asymptomatic infections has a clear potential public health outcome. In a scenario where pregnant women are intermittently screened, we need to know how much more sensitive the HS-RDT is compared to a co-RDT in this population, and whether using a more sensitive test could reduce malaria burden in pregnant women and improve pregnancy outcomes. The testing of pregnant women at their first ANC visit has been identified as a potential sentinel surveillance strategy for monitoring population-level changes in prevalence, but questions remain as to whether this approach accurately captures these trends, and whether an HS-RDT would improve the accuracy of this strategy.

In this article we summarise published and available unpublished data to evaluate the performance of the HS-RDT across different transmission settings and use cases.

## Methods

### Systematic review and data description

**Fig. 1:**
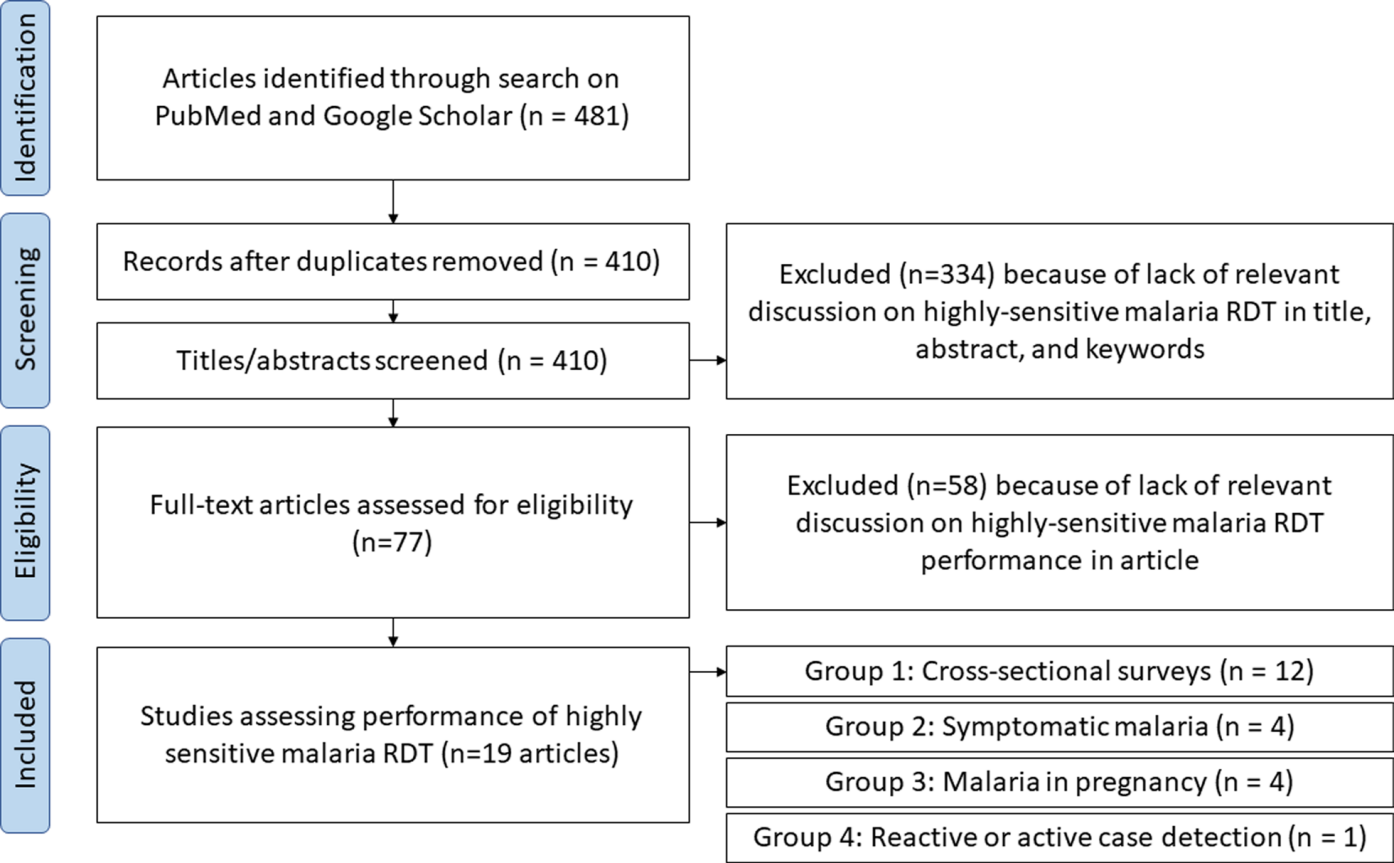
Flow chart of identification of published studies. The breakdown on studies in the ‘included’ section is greater than the total number of studies as several studies fall into two categories

Searches for studies using the Abbott HS-RDT were carried out using PubMed and Google Scholar using the following search terms (“ultra-sensitive” OR “ultra sensitive” OR “ultrasensitive” OR “uRDT” OR “HS-RDT” OR “HS RDT” OR “HSRDT” OR “high-sensitivity RDT” AND “malaria” AND “rapid diagnostic test”) and searching for publications after 2017 (as this was the year the test was launched). This search yielded 481 records, of which 410 were unique records. After screening titles and abstracts, 75 articles were retained for full-text evaluation (Fig. 1). Of these, data were extracted from 18 articles and analysed in this study (Table 1). Studies were retained if they (i) used the HS-RDT (05FK140), (ii) contained performance data with denominators on the HS-RDT and at least one other additional diagnostic method. Investigators of three unpublished studies that were presented at two side meetings on the HS-RDT at the Annual Meeting for the American Society of Tropical Medicine and Hygiene (2017, 2018) were also contacted and agreed to the inclusion of their data in this analysis [9–11].

**Table 1:** Details of published and unpublished studies used in this study

First author, year, reference	Country	Study description	HRP2	PCR/other NAAT method	qPCR	co-RDT
Acquah et al. 2021 [19]	Ghana	Cross-sectional prevalence		x	x	x
Das et al. 2017 [12]	Myanmar, Uganda	Cross-sectional prevalence	x	x	x	x
Druetz et al. 2020 [16]	Haiti	Cross-sectional prevalence				x
Galatas et al. 2020 [20]	Mozambique	Cross-sectional prevalence	x	x		x
Girma et al. 2019 [21]	Ethiopia	Cross-sectional prevalence		x	x	x
Hofmann et al. 2018 [14]	PNG	Cross-sectional prevalence		x	x	x
Landier et al. 2018 [13]	Myanmar	Cross-sectional prevalence	x	x	x	x
Liu et al. 2019 [22]	Myanmar	Cross-sectional prevalence		x		x
Manjurano et al. 2021 [23]	Tanzania	Cross-sectional prevalence		x		x
Mwesigwa et al. 2019 [24]	The Gambia	Cross-sectional prevalence		x		
Owalla et al. 2020 [25]	Uganda	Cross-sectional prevalence, Clinical diagnosis			x	x
Yeung et al. 2020 [15]	Cambodia	Cross-sectional prevalence, Active case detection			x	x
Hartley et al. 2020 [26]	Tanzania	Clinical diagnosis			x	x
Hofmann et al. 2019 [27]	Tanzania	Clinical diagnosis		x	x	x
Plucinski et al. 2017 [28]	Angola	Clinical diagnosis	x			x
Briand et al. 2020 [29]	Benin	Pregnant women		x		
Unwin et al. 2020 [30]	Indonesia	Pregnant women		x		x
Vásquez et al. 2018 [31]	Colombia	Pregnant women		x		x
Vásquez et al. 2020 [32]	Colombia	Pregnant women		x	x	x
Unpublished studies						
Bridges et al. [10]	Zambia	Cross-sectional prevalence, Active case detection		x		x
Saad et al. [11]	Cambodia	Cross-sectional prevalence, Active case detection		x	x	
Bennett et al. [9]	Laos	Cross-sectional prevalence, Active case detection		x		x

The four columns to the right show which other diagnostics were used in each study

Studies were categorised into four groups: Group 1 consisted of studies reporting the prevalence of asymptomatic malaria using the HS-RDT and ideally a molecular NAAT (typically PCR) in cross-sectional population surveys (12 published studies, 3 unpublished studies). Some studies in this group provided multiple prevalence data points as they consisted of multiple population groups [12], used HS-RDTs under both laboratory and field conditions [13], used different PCR methods as the reference standard [14], or used different approaches to sample asymptomatic individuals [15, 16]. This resulted in 20 prevalence data points; of these, all but 2 had PCR as a reference standard, and all but 3 also tested samples using a co-RDT. Group 2 consisted of studies performed in symptomatic individuals presenting for treatment at health facilities (n = 4), although one of these studies only estimated the performance of an HS-RDT based on a hypothetical limit of detection compared to antigen concentrations from febrile patients. Group 3 included studies testing the performance of the test in pregnant women (n = 4), and finally, group 4 included studies using the test in some form of reactive or active case detection (one published study and three unpublished studies). Some studies were included in multiple categories.

Access to full individual-level datasets was available from two studies in Myanmar [13] and Uganda [12]: these were used in the assessment of analytical test performance. The Myanmar dataset consists of data from 1847 specimens from asymptomatic individuals residing in Kayin State, of which 185 have PCR positive *P. falciparum* infections and 66 have mixed *P. falciparum* and *P. vivax* infections. The Uganda dataset consists of data on 607 specimens from asymptomatic individuals residing in Nagongera taken at two time points 3 months apart, of which 249 are PCR positive for *P. falciparum* only and 12 are PCR positive for *P. falciparum* mixed infections. The mean prevalence of PCR-positive infection with any *P. falciparum* is 13.6% (251/1847) in the Myanmar dataset and 43.0% (261/607) in the Uganda dataset. Both sets of samples were also run on the Quansys antigen quantification platform [17, 18] which provides an HRP2 concentration value for each sample. Both studies also tested samples using a co-RDT and quantitative PCR (qPCR). Details of all studies used in this article are shown in Table 1.

### Statistical analyses

Two analyses were conducted to assess the analytical performance of the test using two datasets for which we had individual-level data including HRP2 concentrations. Firstly, for each dataset, the data were split into six categories based on HRP2 concentration (< 1; 1–10; 10–100; 100–1000; 1000–10,000; 10,000–100,000 pg/ml), and the sensitivity of the HS-RDT and co-RDT were calculated for each category based on the proportion of PCR-positive samples in that category that were also positive by each RDT. Secondly, logistic regression models were fitted for (i) the probability of RDT positivity (HS-RDT and co-RDT) as a function of HRP2 concentration and (ii) the probability of RDT positivity (HS-RDT and co-RDT) as a function of parasite density as estimated by qPCR. For the HRP2-based logistic regression model, concentration values below the lower limit of detection (LLOD) were set to 0.05 pg/ml as this is half the lowest LLOD of the two studies, and values above the upper limit of detection (ULOD) were set to the respective ULOD value for each study. For the PCR model, in the Myanmar dataset, samples with discordant PCR positivity results (as samples were analysed twice) were excluded from the analysis, as were samples where the *Plasmodium* species was unable to be identified and samples that were *P. vivax*–only infections. Mixed *P. falciparum*/*P. vivax*, *P. falciparum*–only, and negative samples were retained. There were no non-*falciparum* samples in the Uganda dataset, so all samples were retained. Negative samples were set to the lowest value by the PCR method in each study (0.01 parasites/µl in the Uganda data and 0.0000002 parasites/µl in the Myanmar data). Logistic regression models were fitted using a Hamiltonian Monte Carlo procedure implemented in Stan via the brms package in R [31]. The RDT status (0, 1) was the dependent variable, and HRP2 concentration or parasite density were the independent variables. Informative gaussian priors with a mean of 0 and standard deviation of 3 were used for the model parameters. Two chains were run for each model for 5000 iterations after a burn-in of 2500 iterations. Convergence was visually assessed from the traceplots of both parameters. The fitted line and shaded area show the median and the 95% credible interval from the logit-transformed posterior samples from the linear predictor.

Using summary aggregated data from 12 published and 3 unpublished studies that contain information on cross-sectional prevalence (Table 1), the performance of the HS-RDT in this use case was assessed in three ways: (i) comparing HS-RDT prevalence against PCR prevalence and co-RDT prevalence; (ii) calculating sensitivity of the HS-RDT and the co-RDT, calculated as the proportion of PCR-positive samples that are each positive by each RDT. A binomial logistic regression model is used to assess the relationship between PCR prevalence and sensitivity, and the weighted mean sensitivity is calculated for each test. Lastly, (iii) calculating the ratio of HS-RDT prevalence to co-RDT prevalence for each study where both tests were used, and then calculating an aggregated ratio across all studies. The overall ratio was calculated using a random effects model for meta-analysis with the DerSimonian–Laird method using the ‘rmeta’ package in R [32], and the relative risks are presented. One study [25] only used the HS-RDT and PCR on a non-random subset of samples, namely 25 co-RDT-negative individuals and 25 co-RDT-positive individuals. These numbers were converted to population-level prevalence estimates using methods detailed in Additional file 1.

For the four studies that used the HS-RDT to screen pregnant women, the sensitivity of the HS-RDT and co-RDT was calculated as the proportion of PCR-positive samples that tested positive using each RDT.

## Results

### Sensitivity of the HS-RDT against HRP2 concentration

**Fig. 2:**
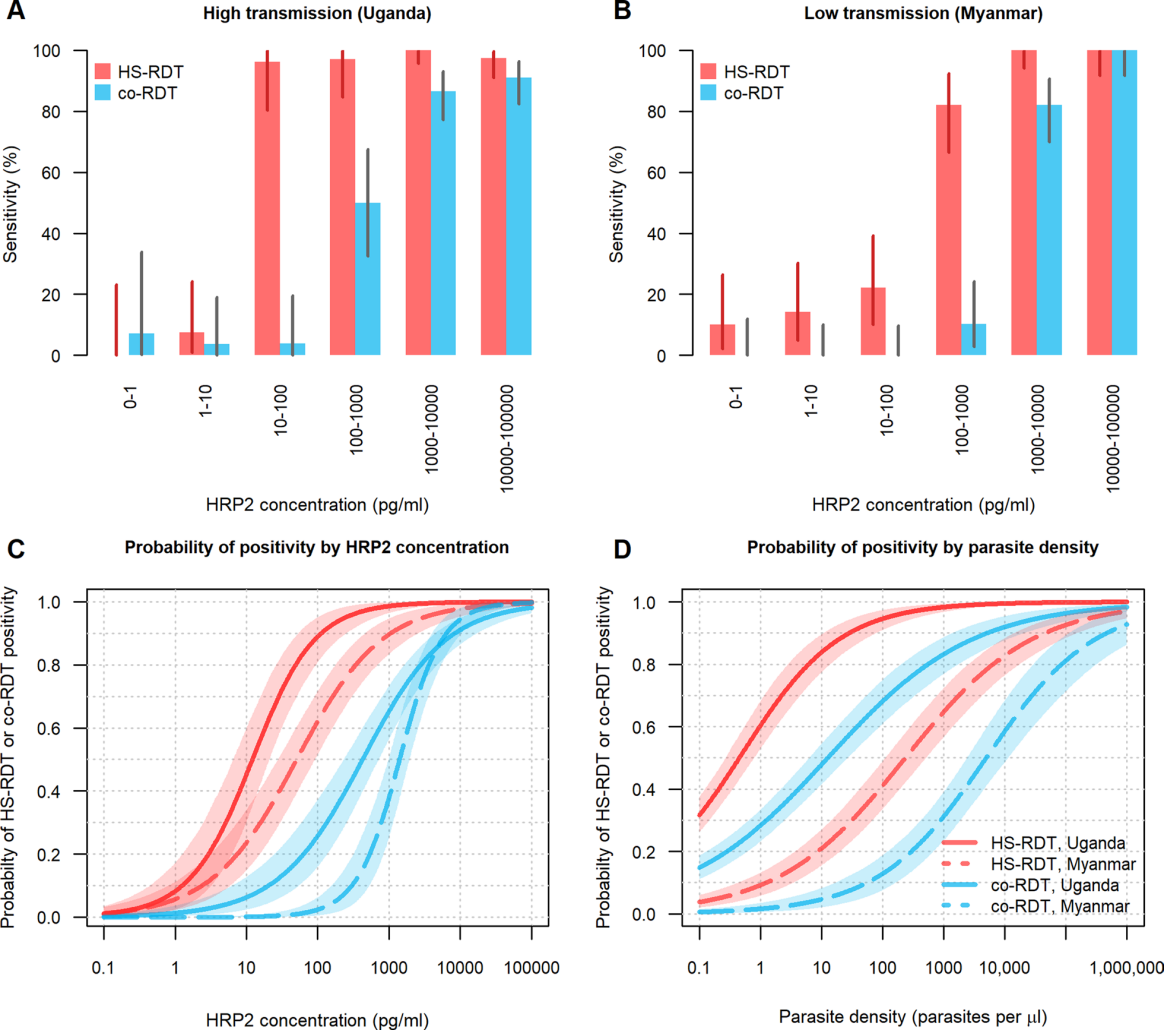
Performance of the HS-RDT against HRP2 concentration in PCR-confirmed specimens. Panels **A** and **B** show the sensitivity of the HS-RDT and co-RDT in samples grouped by different levels of HRP2 for a high-transmission setting (Uganda, **A**) and a low-transmission setting (Myanmar, **B**). Sensitivity is defined as the proportion of PCR-positive samples that are also detected by each RDT. The vertical lines on each bar are 95% binomial confidence intervals for each estimate. Panel **C** shows the probability of HS-RDT (red lines) and co-RDT (blue lines) positivity as a function of HRP2 concentration and panel **D** shows the probability of HS-RDT (red lines) and co-RDT (blue lines) as a function of parasite density by quantitative PCR. The shaded region indicates the 95% credible interval of the model fit

The performance of the HS-RDT on clinical samples based on HRP2 concentration was assessed by calculating the sensitivity amongst samples with different levels of HRP2. In samples with HRP2 concentrations > 1000 pg/ml, the HS-RDT had a mean sensitivity of 99.2% across the high- and low-transmission settings (Fig. 2A and B, respectively) and co-RDT had a sensitivity of > 89.0% in these same samples. The sensitivity of the HS-RDT remained high in samples with HRP2 concentrations 100–1000 pg/ml, with values of 97.1% in Uganda and 82.1% in Myanmar, whereas the sensitivities of the co-RDT were 50.0% and 10.3%, respectively. The sensitivity of the HS-RDT greatly decreased in Myanmar for samples with concentrations of 10–100 pg/ml (sensitivity = 22.2%). In Uganda, sensitivity was still high at this level of HRP2 concentration (sensitivity = 96.2%), but decreased greatly in samples with concentrations of than 1–10 pg/ml (sensitivity = 7.4%). For both settings, the HS-RDT is significantly more sensitive than the co-RDT in samples with HRP2 concentrations that are between 10 and 1000 pg/ml. Figure 2C and D show the probability of positivity either by HRP2 concentration or by parasite density, respectively. The difference in profiles between the Myanmar and Uganda relationships to HRP2 concentration and parasite density may suggest operational differences in the study and differences in the sensitivity of the qPCR method, as these would be anticipated to behave more similarly. The correlation between parasite density by qPCR and HRP2 concentration for both studies is shown in Additional file 4: Fig. S1.

### Cross-sectional prevalence estimates and sensitivity of the HS-RDT

**Fig. 3:**
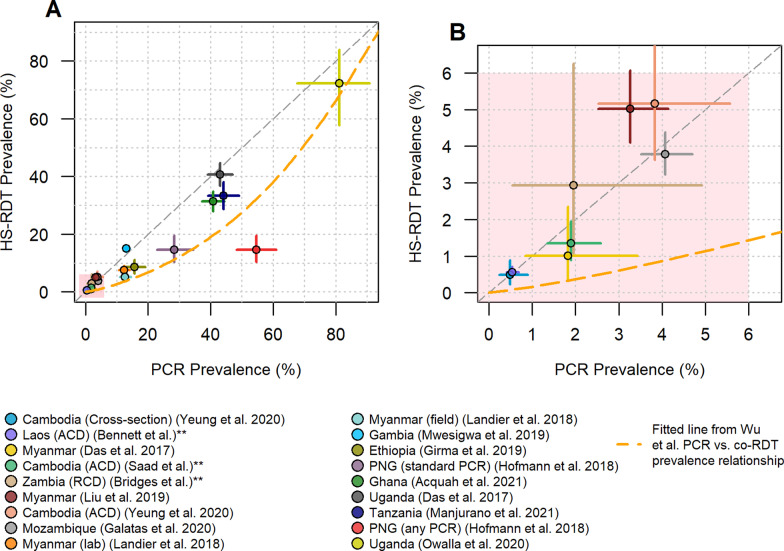
Comparison of PCR prevalence against HS-RDT prevalence. **A** shows all data used in this analysis (n = 18), and **B** shows a zoom-in of the samples with prevalence below 6%. The horizontal and vertical lines from each data point show the binomial confidence intervals associated with the PCR prevalence and HS-RDT prevalence estimates, respectively. The orange dashed line shows the fitted relationship derived from a previous meta-analysis of PCR and co-RDT prevalence surveys [3]. The grey diagonal line shows the x = y equivalence line between the HS-RDT and PCR. Additional details are provided on the sample type where necessary. **Unpublished studies. *PNG* Papua New Guinea, *RCD* reactive case detection, *ACD* active case detection

Comparing the prevalence estimates obtained using an HS-RDT and PCR in cross-sectional prevalence surveys (Fig. 3), we see that in all but four of the studies the prevalence falls below the diagonal x = y line, indicating that generally HS-RDT prevalence is lower than PCR prevalence. In the very low-transmission settings, all data points fall close to this line (Fig. 3B), showing good concordance between the two diagnostics. A fitted line from a previously published meta-analysis looking at PCR versus co-RDT prevalence estimates [3] was added to the plots. All the HS-RDT prevalence estimates apart from one lie above line, suggesting the HS-RDT is a more accurate tool than a co-RDT for measuring prevalence across a broad range of transmission settings. In all studies, there is some discordance between the individuals that test positive for PCR and those that test positive by HS-RDT due to the tests measuring two different analytes.

One study allows observation of the impact of the PCR method used on the comparative prevalence between the HS-RDT and PCR [14]. In Fig. 3, the red and mauve data points are from the same samples from Papua New Guinea (PNG); however, the mauve point shows prevalence as estimated using a standard PCR assay whereas the red point shows prevalence obtained by assuming any one of three PCR assays being positive means the sample is positive, including an ultra-sensitive technique that they found to be 10 × more sensitive than their standard assay [33]. Another study in Myanmar enabled comparison of the performance of the HS-RDT under controlled laboratory settings (orange) versus field conditions (turquoise) [13]; while the prevalence estimate is lower for the HS-RDT run in field conditions versus laboratory conditions, the same drop was also observed with the co-RDT.

**Fig. 4:**
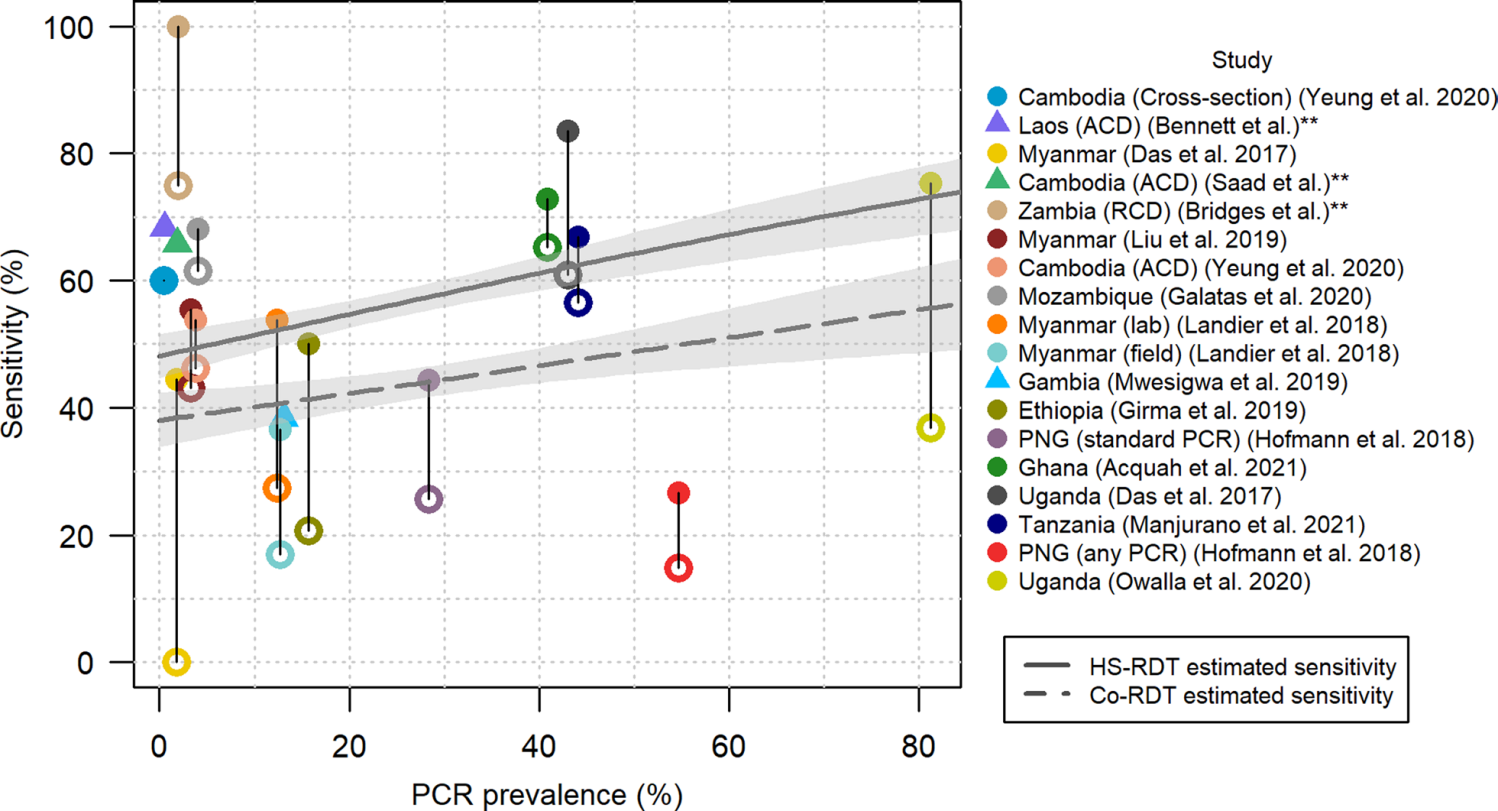
Sensitivity of the HS-RDT and co-RDT against PCR prevalence. The filled circles and triangles show the sensitivity of HS-RDT. The unfilled circles joined to the filled circles by a line show the sensitivity of the co-RDT in the same study, if this test was used. The triangles indicate studies where a co-RDT was not used. PCR is the gold-standard diagnostic against which sensitivity is calculated. The solid grey and dashed grey lines show the fit from a binomial generalised linear model of the relationship between PCR prevalence and sensitivity of the HS-RDT and co-RDT respectively. **Unpublished studies. *RCD* reactive case detection, *ACD* active case detection

The sensitivity of the HS-RDT and the co-RDT in all the studies (where available) was plotted against PCR prevalence in Fig. 4. A binomial regression model was used to explore whether there was a relationship between PCR prevalence and sensitivity for each RDT. For both the HS-RDT and the co-RDT, there is a statistically significant positive relationship between PCR prevalence and sensitivity (p < 0.05). The weighted mean sensitivity of the HS-RDT across all studies was estimated to be 56.1% (95% confidence interval [CI] from weighted t-test = 46.9–65.4%) compared to 44.3% (95% CI 32.6–56.0%) for the co-RDT. The vertical segments join the sensitivity values from the same study—the length of this segment indicates the percentage point increase in sensitivity of the HS-RDT compared to the co-RDT.

**Fig. 5:**
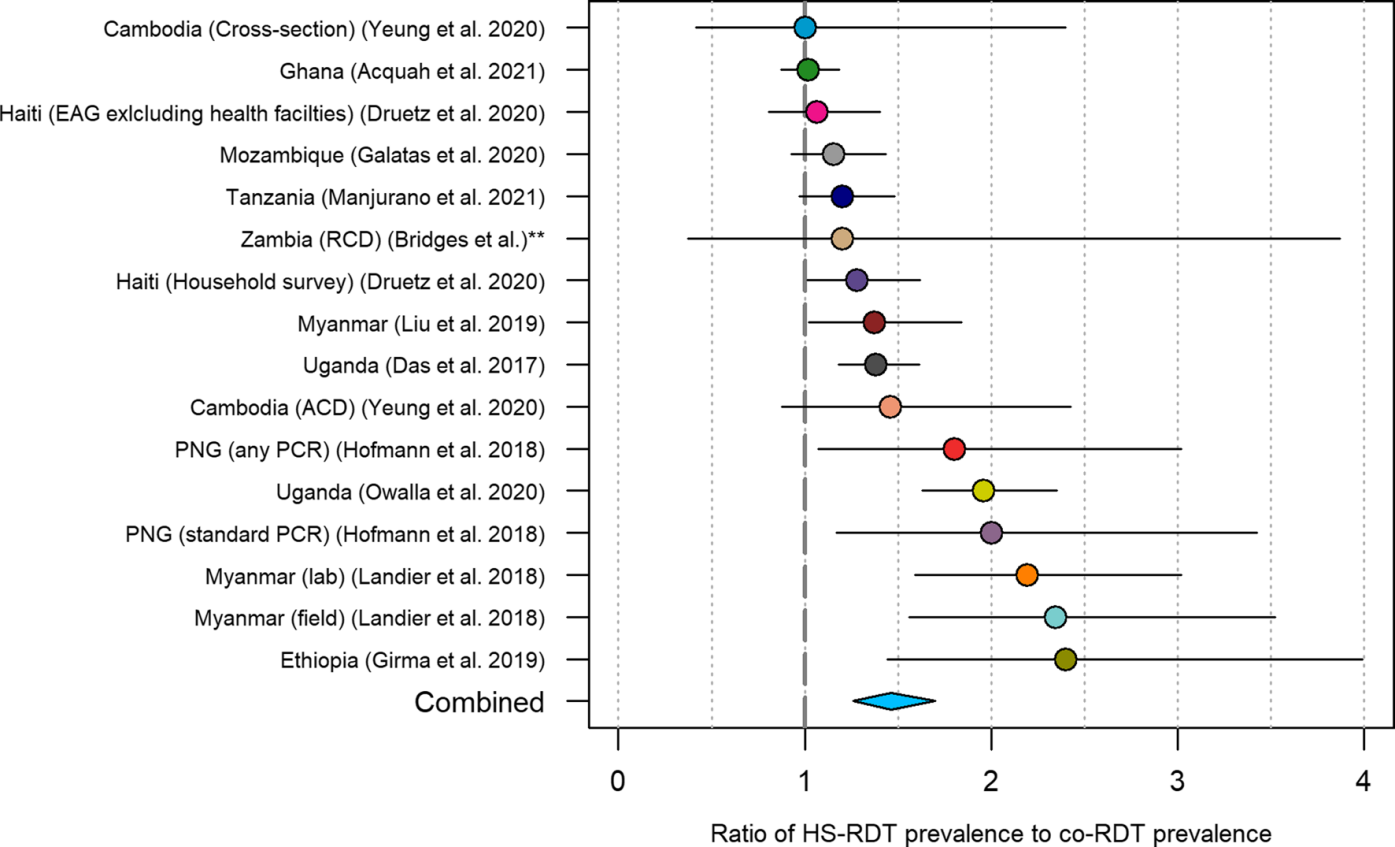
Ratio of HS-RDT prevalence to co-RDT prevalence in 16 surveys from 15 studies. The circles show the estimated ratio, and the horizontal lines show the associated binomial 95% confidence intervals. The centre of the blue diamond shows the weighted mean estimated ratio (1.46) and the horizontal extents indicate the 95% confidence interval (1.26–1.70). **Unpublished studies. *EAG* easy access group, *RCD* reactive case detection, *ACD* active case detection

To explore the increase in HS-RDT prevalence estimates compared to those from using a co-RDT, we plotted the ratio of these values in all studies where both tests were used (Fig. 5). The weighted mean estimated ratio of HS-RDT prevalence to co-RDT prevalence is 1.46 (95% CI 1.26–1.70). This means that prevalence estimated using an HS-RDT will be on average 46% higher than if using a co-RDT.

Positive and negative predictive values (PPV and NPV) for the HS-RDT and co-RDT are shown for all studies where data are available in Additional file 2. The unweighted mean PPV across all studies where data were available is 0.75 for the HS-RDT and 0.79 for the co-RDT. The unweighted mean NPV value is 0.91 for the HS-RDT and 0.89 for the co-RDT.

### Use for clinical case management

Conventional RDTs are considered to be effective for *P. falciparum* clinical case management, with high sensitivity against clinical infection [34, 35] that is associated with high parasite densities and HRP2 concentrations. Here we review evidence on the diagnostic performance of the HS-RDT in a clinical setting.

One study has retrospectively evaluated the usefulness of an HS-RDT for clinical diagnosis and fever management compared to a co-RDT [27]. Frozen blood samples from 3000 children and 515 adults presenting with fever to an outpatient clinic in Dar es Salaam, Tanzania, were tested using a co-RDT, an HS-RDT, by ultra-sensitive qPCR, and by enzyme-linked immunosorbent assay (ELISA) to estimate HRP2 concentration. Out of 309 children testing positive by qPCR, 226 (73.1%) and 230 (74.4%) were also positive by co-RDT and HS-RDT, respectively, and out of 48 adults testing positive by qPCR, these values were 35 (72.9%) and 37 (77.1%). Four children and 0 adults were co-RDT positive and qPCR negative, and 9 children and 0 adults were HS-RDT positive and qPCR negative. Of individuals positive for HRP2 by ELISA, 83.1% were positive by a co-RDT and 86.5% were positive by the HS-RDT. This study was conducted in a very low-transmission setting; test positivity rate among febrile individuals (by co-RDT) was only 7.7% in children and 6.8% in adults, which would suggest an even lower population-level prevalence. Therefore, few individuals presenting with non-malarial fevers would be expected to be co- or HS-RDT positive due to having asymptomatic infections or having been recently treated for a malaria infection (and having residual HRP2).

A second study was carried out in the same location [26] and tested 2801 febrile paediatric outpatients. Of the 274 that were PCR positive, 198 and 201 were detected by a co-RDT and an HS-RDT respectively, giving sensitivity values of 72.2% and 73.4%. Furthermore, this study compared the health outcomes up to 28 days after presenting at the health facility with fever. There was no evidence that individuals with PCR-positive, co-RDT-negative infections had worse outcomes compared to PCR-negative individuals in terms of clinical failures (e.g. developing of severe symptoms, having persistent symptoms after 7 days, clinical pneumonia) or secondary hospitalisations.

A study based in Angola measured the HRP2 concentration of outpatients attending a health facility using the multiplex bead assay [28]. The impact of a hypothetical HS-RDT with a LLOD of 200 pg/ml was then estimated by calculating the proportion of individuals that would have HRP2 concentrations above this threshold. They found that 81% of febrile individuals with detectable HRP2 were detected by a co-RDT and an additional 10–20% of cases would have been identified using this hypothetical HS-RDT. In addition, 52 and 77% of HRP2-positive afebrile individuals were detected using a co-RDT in two separate sites, and an additional 50–60% of individuals would have been detected with the hypothetical HS-RDT.

In Uganda, a study was conducted where the HS-RDT was used alongside a conventional RDT and microscopy for testing febrile children under the age of 5 [25]. During 475 clinic visits, positivity by the HS-RDT was 55.2%, by a co-RDT it was 53.5%, and by microscopy it was 40.6%. The HS-RDT yielded only marginally higher positivity leading to the detection of an additional eight more individuals compared to a co-RDT. The co-RDT and HS-RDT each detected 61 and 69 more cases than microscopy.

### Use in screening pregnant women

Four published studies were identified that looked at the performance of the HS-RDT in pregnant women. A study in Benin [29] tested 942 samples in 327 women in the first and third trimesters and at delivery. They found that the overall positivity of the HS-RDT was 16.2% compared to 11.6% by a co-RDT and 18.3% by PCR. Based on 172 PCR-positive samples across all stages of pregnancy, the sensitivity of the HS-RDT was 60.5% compared to 44.2% by a co-RDT. The difference was even more pronounced during the first trimester, where sensitivity by HS-RDT was 57.0% compared to 38.3% by co-RDT.

The authors considered the potential clinical impact of treating women that are positive by HS-RDT but negative by co-RDT (i.e., individuals that would only be detected if an HS-RDT was used) by conducting a multivariate analysis to assess the impact of diagnostic status on maternal and birth outcomes. Individuals in this category (HS-RDT+, co-RDT−) have a 3.4 times higher risk of maternal anaemia during pregnancy compared to uninfected (PCR-negative) women [29]. Co-RDT-positive women had a two times higher risk of anaemia compared to uninfected women. Both these effects were statistically significant, but the difference between them was not. There was a 5.3 times higher risk of low birthweight in the HS-RDT+, co-RDT− group and a 2.3 times higher risk in the co-RDT+ group compared to the uninfected group, but both effects were nonsignificant.

A study in Colombia [31] tested 737 peripheral and placental samples using the HS-RDT as well as light microscopy, nested PCR, a *Pf*-only RDT and a *Pf*/*Pv* RDT. Among all samples, the HS-RDT performed comparably to the best performing co-RDT (sensitivity of 85.7% compared to 82.8%). The authors also disaggregated the data by whether each woman was symptomatic at the time the sample was taken (defined as fever with an axillary temperature ≥ 37.5 °C or history of fever within the last 3 days). The sensitivity was high and exactly the same using all diagnostics among symptomatic women (85.7%, n = 61). Among asymptomatic women (n = 649), sensitivity using the HS-RDT was 71.4% compared to 64.3% with the best performing co-RDT and 50% by light microscopy, although none of the differences were statistically significant.

A second study in Colombia tested 858 pregnant women attending an ANC clinic [32] with an HS-RDT, a co-RDT, microscopy, loop-mediated isothermal amplification (LAMP), and both nested PCR and quantitative reverse transcription PCR (qRT-PCR). The overall prevalence of *P. falciparum* infection among the participants was 4.5% by the most sensitive diagnostic, qRT-PCR. Using this as the standard reference, the sensitivities of the HS-RDT, co-RDT, microscopy, and LAMP were 64.1%, 53.8%, 59.0%, and 89.7%, respectively. There were four women that were positive by HS-RDT and negative by co-RDT—they all had parasite densities < 100 parasites/µl.

Finally, the performance of the HS-RDT was retrospectively tested against reconstituted stored samples from pregnant women in Indonesia [30]. Based on 158 samples positive for *Pf* by PCR, the sensitivity of the HS-RDT was 19.6% compared to 22.8% using a co-RDT, indicating that in this population there was no improvement in sensitivity.

### Active case detection

One study in Cambodia used the HS-RDT for both reactive and proactive case detection [15]. In the reactive case detection, the households, high-risk neighbours, and co-travellers of passively detected symptomatic ‘index cases’ were screened with an HS-RDT, a co-RDT, and qPCR. In proactive case detection, all high-risk individuals in villages with a high total number of malaria cases were proactively screened. High-risk individuals were defined as those reporting fever in past 48 h or having slept in the forest in the past month. A total of 678 individuals were tested as part of both reactive and proactive case detection. Only 26 individuals were PCR positive, of which 12 were detected by a co-RDT and 14 by the HS-RDT. Furthermore, 12 and 21 PCR-negative individuals were co-RDT and HS-RDT positive, respectively.

A second study in Cambodia used the HS-RDT to conduct active monthly screening of asymptomatic high-risk forest and plantation workers in 11 villages [11]. In this very low-transmission setting, the HS-RDT had a sensitivity of 66.6% with PCR as the gold standard. The maximum parasite density amongst individuals with false negative HS-RDT results was less than 400 parasites/µl and the median parasite density was only 2 parasites/µl, indicating the test missed mostly low-density infections.

In Zambia, the HS-RDT was used in a reactive focal test and treat study where households of index cases detected at a facility were tested with both a co-RDT and HS-RDT [10] (HS-RDT data unpublished). Prevalence was very low in this trial, with only 4/205 individuals testing positive by PCR. The HS-RDT detected all four of these individuals and the co-RDT only detected three.

In another very low transmission setting in Laos [9], the HS-RDT was used in active case detection (HS-RDT data unpublished). Of 11,771 individuals tested by PCR and HS-RDT there were only 63 and 66 positives, respectively. Of the 63 PCR positive samples, 43 of them were also positive by the HS-RDT.

## Discussion

The performance of any RDT depends on both the limit of detection of the test and on the distribution of target analyte in the populations where it is being used. In the specific case presented in this review, a test with a given LOD will have lower diagnostic sensitivity in infected populations with lower HRP2 concentrations and higher diagnostic sensitivity in populations with higher HRP2 concentrations. The performance of a test (HS-RDT) with a lower LOD than another test (co-RDT) will be more resilient to fluctuations in the target analyte, in this case HRP2 distribution in a given population. Furthermore, the improvement in sensitivity for a test with a lower LOD will depend on the proportion of the population whose analyte concentrations fall between the LOD of the new test and the LOD of the conventional test.

In asymptomatic cross-sectional surveys, the sensitivity of the HS-RDT in asymptomatic populations is estimated to be 56.1% compared to 44.3% with a co-RDT with PCR as the reference standard. We found a positive relationship between PCR prevalence and the sensitivity of the HS-RDT, indicating that it may perform relatively better in high-transmission settings. This is consistent with evidence that parasite densities are higher in high-transmission settings [4]. The HS-RDT is estimated to detect on average 46% more infections than a co-RDT (Fig. 5). The results presented here show that the HS-RDT consistently outperforms co-RDTs when used for cross-sectional surveys.

For clinical diagnosis, the incremental benefit of using the HS-RDT compared to a co-RDT to test febrile individuals appears to be marginal, with all studies reporting a small number of additional PCR-positive cases detected with the more sensitive test. This is likely because the antigen concentrations of individuals with febrile malaria are higher than in asymptomatic populations and are in the range where the co-RDT already performs well (Fig. 2A, B). The HS-RDT did detect more false positives (PCR negative, HS-RDT positive) compared to the co-RDT, highlighting the importance of proper clinical management to investigate a range of causes of the fever for RDT-positive individuals. This is particularly relevant in settings where proper diagnostic skills to identify other causes of fever may be limited, as is often the case among community health workers and basically trained health staff. This is especially a concern in high-transmission areas with a high probability of having recent malaria episodes with persistent antigens. Conversely, the HS-RDT may also detect new infections sooner [12] which is a clear benefit in highly susceptible populations such as infants. The risk–benefit of a more sensitive test for malaria case management needs to be better understood.

For screening pregnant women for the malaria, the results here indicate that the HS-RDT may be more sensitive than a co-RDT for detecting malaria in pregnant women in all but one study, and that the additional infections detected by the HS-RDT may have clinical significance for the mother and child. However, only the data aggregated across all stages of pregnancy from the Benin trial [29] produced a statistically significant result. Currently, WHO recommends IPTp in areas with moderate to high transmission. However, there is widespread resistance to sulfadoxine-pyrimethamine, the antimalarial used in this intervention [36]. This has led to calls for a screening-based approach where women are screened with an RDT regardless of symptoms and are treated with a more efficacious antimalarial if positive. Initial trials of this approach using a co-RDT have had mixed results and as such this intervention is not recommended by WHO [37–41]. However, it has been considered that a more sensitive screening tool could improve the effectiveness of this approach [6]. Additionally, trials have investigated the safety of giving antimalarials to women in the first trimester, which if recommended by WHO, would be more impactful if a highly sensitive and specific RDT was available in antenatal clinics.

The Senegal National Malaria Control Programme has recently started to evaluate the use the HS-RDT in reactive case detection strategy. However, there is currently limited direct evidence on the added value of an HS-RDT in active case detection. Results presented here indicate that the HS-RDT is more sensitive than the co-RDT in asymptomatic individuals, suggesting that its use in active case detection would likely improve the effectiveness of the intervention compared to using a co-RDT.

The HS-RDT has a more limited stability claim than most co-RDTs; it claims storage stability to 30 °C versus 40 °C for most of the WHO prequalified RDTs. It also has a more restricted shelf life of 12 months, compared to 24 months for other prequalified co-RDTs [42]. A survey of many of the investigators of the studies described here suggests that many were aware of these limitations and managed them, but many studies also did not track temperature exposure. There is a possibility that HS-RDTs in some of the studies were compromised, perhaps leading to reduced incremental benefits of the HS-RDT over the co-RDT. Some studies compared the performance of the tests on frozen samples and in one case reconstituted specimens by combining retrospectively blood pellets with plasma [30]. The impact of this on the performance of any given RDT is not properly characterized and may have influenced some of the study results. Conversely, different studies used different co-RDTs, which will have had different LOD for HRP2 and consequently the difference in comparative test performance may be affected by this. The LOD of HRP2-based RDTs has been shown to vary up to fourfold depending on the product and parasite culture strain investigated [43]. This, together with lot-to-lot performance variations, could confound the gain in clinical sensitivity observed between the HS-RDT and co-RDTs. Furthermore, PCR is typically used as the reference assay for assessing the performance of a new RDT, however, there is known to be large variation in the sensitivity of different PCR assays. For example, a highly sensitive PCR assay may detect very low density infections that also have very low levels of HRP2. RDTs may miss these infections and as a result have lower estimated sensitivity values. An investigation of the impact of PCR sensitivity is presented in Additional file 3. Results indicate that the HS-RDT sensitivity estimates were relatively robust to the PCR assay sensitivity, however the co-RDT performed significantly worse in studies where a sensitive PCR assay was used.

The Alere™/Abbott Malaria Ag P.f RDT is the first malaria RDT that has been launched with the claim of being ‘highly sensitive’. However, incremental improvements to RDT performance have been continuous and ongoing since development of these tools began in the 1990s. The HS-RDT has been submitted to unprecedented evaluation, with dozens of peer-reviewed articles published since its launch in 2017. The HS-RDT has catalysed a better understanding of how to evaluate RDTs and even the development and adoption of quantitative antigen tests to support their evaluation [17, 18, 43, 44]. The lessons learnt with the HS-RDT will help more quickly assess the implications of improvements in LOD for other tests in terms of sensitivity in different population groups and use cases.

## Conclusions

The evidence presented here indicates that the HS-RDT is more sensitive than a conventional RDT for testing asymptomatic populations and could provide a marginal improvement in clinical diagnosis and screening pregnant women. Provided proper clinical management of fevers is possible, and the HS-RDT has comparable costs, heat stability and shelf-life to co-RDTs, there is no evidence to suggest it would have any negative impacts in terms of malaria misdiagnosis if it were widely used in all four population groups explored here.

## Supplementary Information

**Additional file 1.** Estimating population-level prevalence by HS-RDT and PCR in Owalla et al.

**Additional file 2.** Positive and negative predictive values for the HS-RDT and co-RDT.

**Additional file 3.** Sub-analyses on the potential biases associated with PCR sensitivity.

**Additional file 4.** Correlation between parasite density and HRP2 concentration, and positivity by HS-RDT and co-RDT.

## Data Availability

The published datasets analysed, and all code written during the current study are available from https://github.com/PATH-Global-Health/HS-RDT_analysis. Individual-level datasets from Myanmar and Uganda are available from the corresponding author on request.

## Abbreviations

ACD: Active case detection
ANC: Antenatal care
CI: Confidence interval
Co-RDT: Conventional RDT
ELISA: Enzyme-linked immunosorbent assay
HRP2: Histidine-rich protein 2
HS-RDT: Highly sensitive rapid diagnostic test
LAMP: Loop-mediated isothermal amplification
LLOD: Lower limit of detection
LOD: Limit of detection
NAAT: Nucleic acid amplification–based tests
NPV: Negative predictive value
PCR: Polymerase chain reaction
PNG: Papua New Guinea
PPV: Positive predictive value
qPCR: Quantitative polymerase chain reaction
qRT-PCR: Quantitative reverse transcription polymerase chain reaction
RCD: Reactive case detection
RDT: Rapid diagnostic test
ULOD: Upper limit of detection
WHO: World Health Organization
