# Supplementary material for: Performance and utility of more highly sensitive malaria rapid diagnostic tests
Source: BMC Infect Dis. 2022 Feb 4;22:121. doi: 10.1186/s12879-021-07023-5 (PMC8815208; doi:10.1186/s12879-021-07023-5)
Supplement: Supplementary file 1 — Additional file 1. Estimating population-level prevalence by HS-RDT and PCR in Owalla et al. [file 12879_2021_7023_MOESM1_ESM.docx]

**Additional file 1**

*Estimating population-level prevalence by HS-RDT and PCR in Owalla et al. [37]*

In this study, the HS-RDT and PCR were only conducted on a subselection of 25 positive and 25 negative samples on the co-RDT (CareStart), therefore we needed to estimate the population-level prevalence by HS-RDT and PCR based on this biased sample.

|  | Co-RDT-positive samples | Co-RDT-negative samples |
| --- | --- | --- |
| PCR positive | 23/25 | 18/25 |
| HS-RDT positive | 24/25 | 14/24* |

*HS-RDT not run on one co-RDT-negative sample.

Additionally, 36.9% of the total population sampled (n = 1705) were co-RDT positive.

Therefore, we estimate PCR prevalence in the population as:

PCR prevalence = (proportion of the population co-RDT positive x PCR positivity in co-RDT-positive samples) + (proportion of the population co-RDT negative x PCR positivity in co-RDT-negative samples)

= 0.369 x (23/25) + (1 – 0.369) x (18/25) = 0.794

Similarly, for the HS-RDT, prevalence is calculated as:

HS-RDT prevalence = (proportion of the population co-RDT positive x HS-RDT positivity in co-RDT-positive samples) + (proportion of the population co-RDT negative x HS-RDT positivity in co-RDT-negative samples)

= 0.369 x (24/25) + (1 – 0.369) x (14/24) = 0.722

The prevalence values were used in the analyses, assuming denominators of 50 to provide confidence intervals.
