# Supplementary material for: Performance and utility of more highly sensitive malaria rapid diagnostic tests
Source: BMC Infect Dis. 2022 Feb 4;22:121. doi: 10.1186/s12879-021-07023-5 (PMC8815208; doi:10.1186/s12879-021-07023-5)
Supplement: Supplementary file 2 — Additional file 2. Positive and negative predictive values for the HS-RDT and co-RDT. [file 12879_2021_7023_MOESM2_ESM.docx]

**Additional file 2**

*Positive and negative predictive values for the HS-RDT and co-RDT*

The positive predictive value (PPV) was calculated using the following equation:

*PPV = number of true positives / (number of true positives + number of false positives)*

And the negative predictive value (NPV) was calculated as follows:

*NPV = number of true negatives / (number of true negatives + number of false negatives)*

Figures S1 and S2 show these values for the HS-RDT and the co-RDT for studies where are the required data were available. The values from each study for each RDT are joined using a vertical line to ease comparison of the PPV or NPV between the tests for each study.

The unweighted mean PPV across all studies is 0.75 for the HS-RDT and 0.79 for the co-RDT, and weighting by the number of PCR positives in the study, the mean PPV = 0.74 for the HS-RDT and 0.87 for the co-RDT.

The unweighted mean NPV across all studies is 0.91 for the HS-RDT and 0.87 for the co-RDT. Weighting by the number of PCR negatives in the study, the mean NPV = 0.97 for the HS-RDT and 0.94 for the co-RDT.


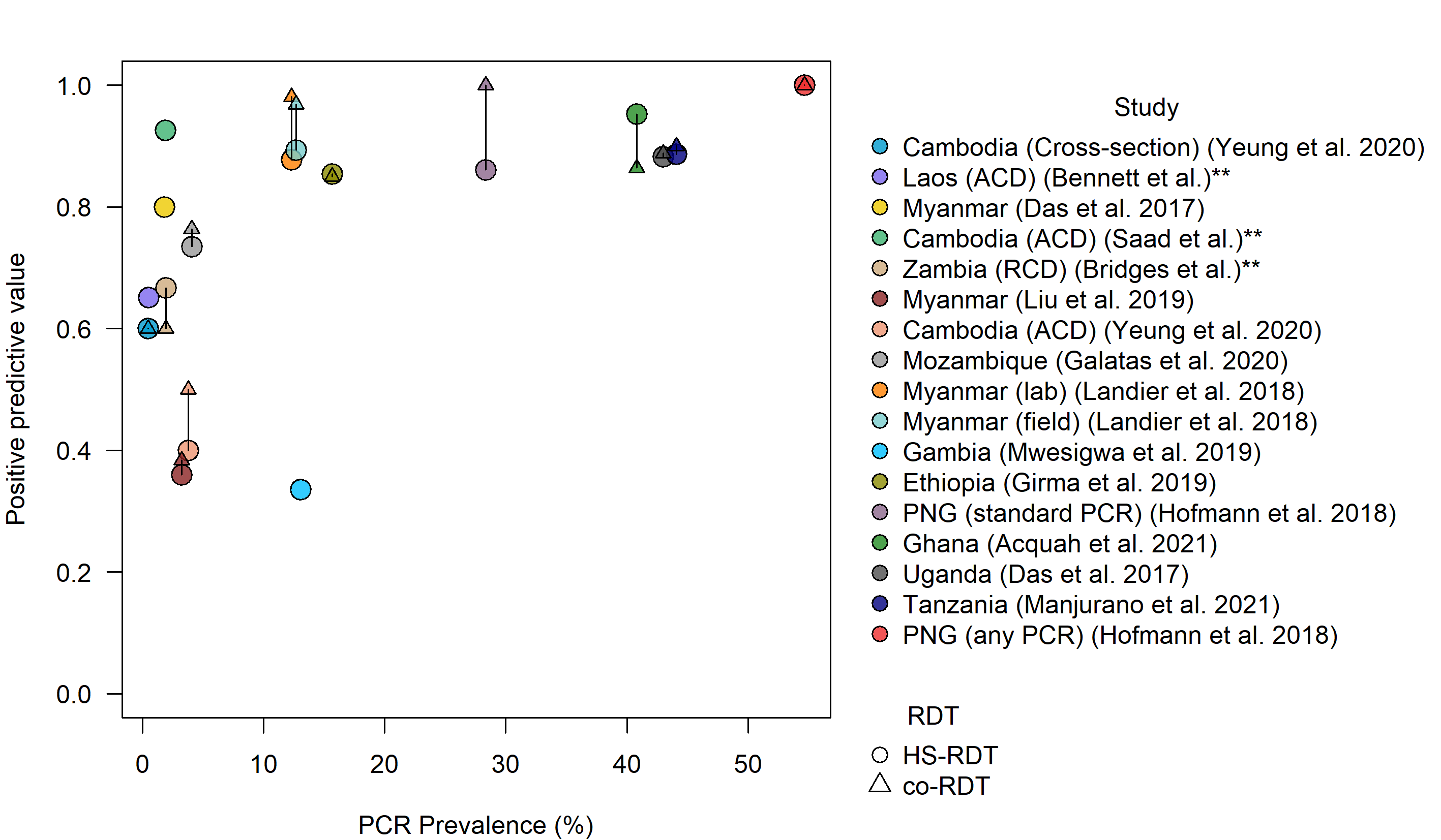


*Figure S2: Positive predictive value for the HS-RDT (circles) and co-RDT (triangles) for each study.*


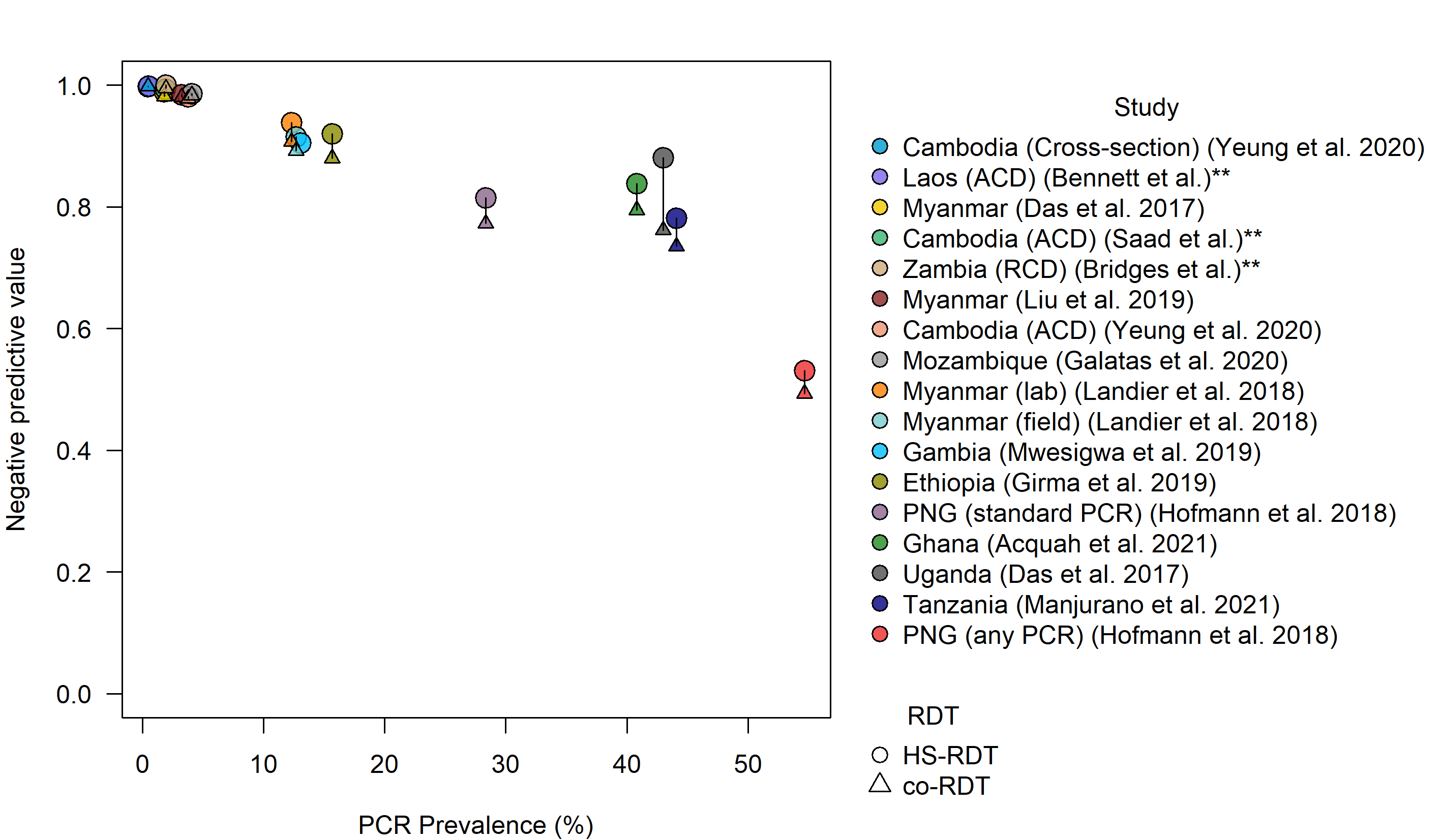


*Figure S3: Negative predictive value for the HS-RDT (circles) and co-RDT (triangles) for each study.*
