# Supplementary material for: Performance and utility of more highly sensitive malaria rapid diagnostic tests
Source: BMC Infect Dis. 2022 Feb 4;22:121. doi: 10.1186/s12879-021-07023-5 (PMC8815208; doi:10.1186/s12879-021-07023-5)
Supplement: Supplementary file 3 — Additional file 3. Sub-analyses on the potential biases associated with PCR sensitivity. [file 12879_2021_7023_MOESM3_ESM.docx]

**Additional file 3**

To investigate the potential biases in the PCR method used, we classified studies as either using a ‘normal’ or a ‘sensitive’ PCR method, based on whether the assay was able to detect below 0.1 parasites per microlitre (table S1)

| First author, year, reference | Country | PCR sensitivity |
| --- | --- | --- |
| Acquah et al. 2021 (19) | Ghana | Normal |
| Das et al. 2017 (12) | Myanmar, Uganda | Sensitive |
| Galatas et al. 2020 (20) | Mozambique | Normal |
| Girma et al. 2019 (21) | Ethiopia | Sensitive |
| Hofmann et al. 2018 (14) | PNG | Both sensitive and normal |
| Landier et al. 2018 (13) | Myanmar | Sensitive |
| Liu et al. 2019 (22) | Myanmar | Normal |
| Manjurano et al. 2021 (23) | Tanzania | Normal |
| Mwesigwa et al. 2019 (24) | The Gambia | Normal |
| Owalla et al. 2020 (25) | Uganda | Sensitive |
| Yeung et al. 2020 (15) | Cambodia | Sensitive |
| Hartley et al. 2020 (26) | Tanzania | Sensitive |
| Hofmann et al. 2019 (27) | Tanzania | Sensitive |
| Briand et al. 2020 (29) | Benin | Normal |
| Unwin et al. 2020 (30) | Indonesia | Normal |
| Vásquez et al. 2018 (31) | Colombia | Normal |
| Vásquez et al. 2020 (32) | Colombia | Sensitive |
|  |  |  |
| Unpublished studies |  |  |
| Bridges et al. (10) | Zambia | Normal |
| Saad et al. (11) | Cambodia | Unknown |
| Bennett et al. (9) | Laos | Normal |

*Table S1: Defining the estimated sensitivity of the PCR in each study.*

The relationship between PCR prevalence and HS-RDT and co-RDT sensitivity is then re-estimating, now using ‘PCR sensitivity’ as a factor in the generalised linear model. The model now has three predictive variables: PCR prevalence, PCR sensitivity, and an interaction term (PCR prevalence * PCR sensitivity).

Figure S4 shows the output of this model. The estimated sensitivity of the HS-RDT is not significantly affected by the PCR sensitivity (comparing the solid pink and solid grey lines). However, the estimated sensitivity of a co-RDT is significantly higher when using a PCR assay with ‘normal’ sensitivity compared to when using a more sensitive PCR assay (comparing the dashed pink and dashed grey lines). This is potentially reflecting the fact that more sensitive PCR assays are detecting a lot of infections with very low parasite densities and thus lower HRP2 concentrations, likely at levels that a co-RDT is unable to detect. However, more data are needed to fully understand the importance of the PCR assay sensitivity in the evaluation of novel malaria RDTs.


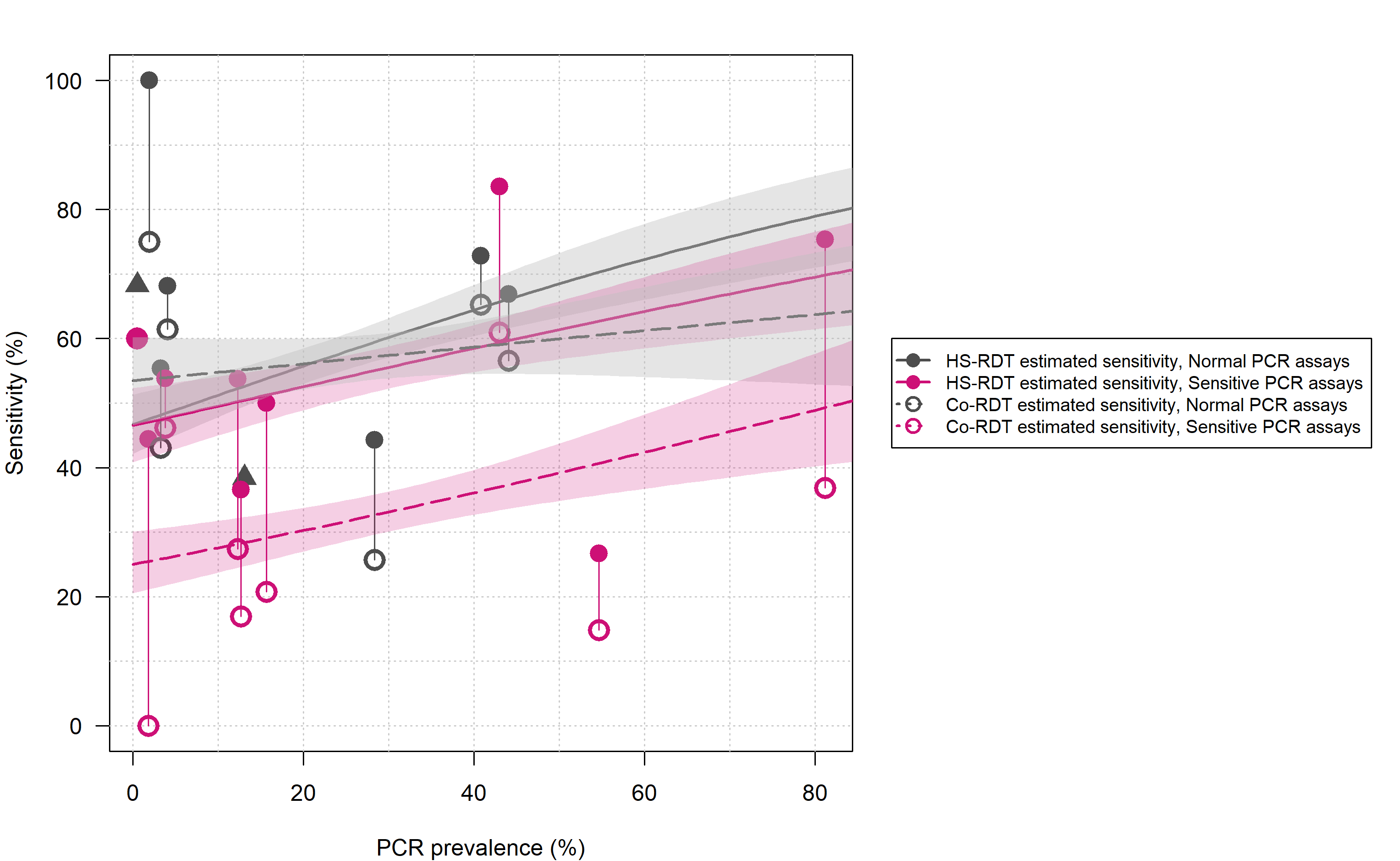


*Figure S4: Modelled sensitivity and 95% confidence intervals for the HS-RDT (solid lines) and co-RDT (dashed lines) with different PCR assay sensitivities (normal - grey, sensitive - pink).*
