# Supplementary material for: Performance and utility of more highly sensitive malaria rapid diagnostic tests
Source: BMC Infect Dis. 2022 Feb 4;22:121. doi: 10.1186/s12879-021-07023-5 (PMC8815208; doi:10.1186/s12879-021-07023-5)
Supplement: Supplementary file 4 — Additional file 4. Correlation between parasite density and HRP2 concentration, and positivity by HS-RDT and co-RDT. [file 12879_2021_7023_MOESM4_ESM.docx]

**Additional Fig. S1**

*Correlation between parasite density and HRP2 concentration, and positivity by HS-RDT and co-RDT.*


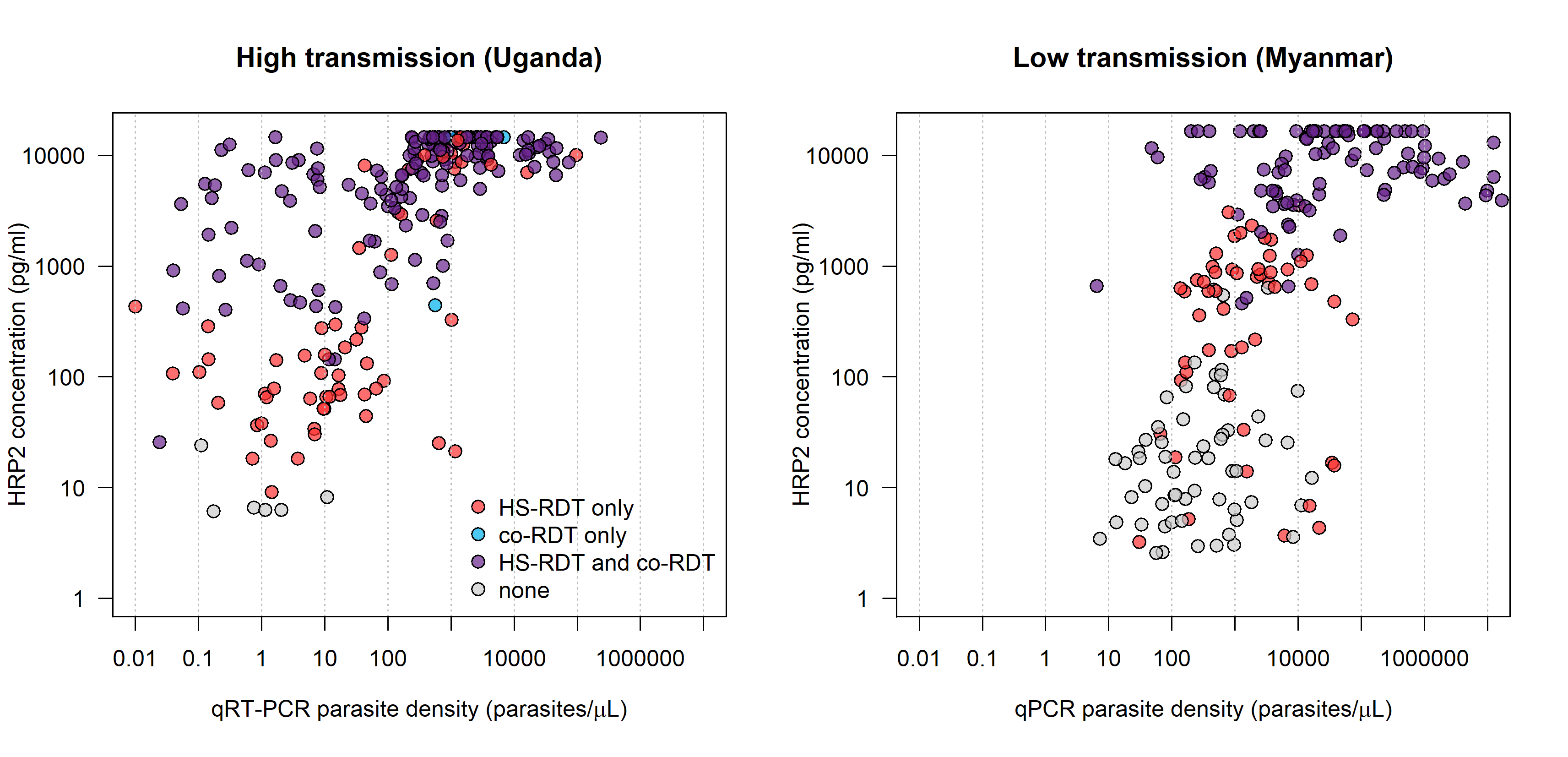


*Panels (****A****) and (****B****) show the relationship between parasite density and HRP2 concentration in a high-transmission setting (Uganda,* ***A****) and a low-transmission setting (Myanmar,* ***B****). Each point is coloured according to whether the sample was positive by HS-RDT only, co-RDT only, both RDTs, or neither RDT.*
